# *ABCB1* Polymorphisms and Drug-Resistant Epilepsy in a Tunisian Population

**DOI:** 10.1155/2019/1343650

**Published:** 2019-12-02

**Authors:** Malek Chouchi, Hedia Klaa, Ilhem Ben-Youssef Turki, Lamia Hila

**Affiliations:** ^1^Tunis El Manar University, Department of Genetic, Faculty of Medicine of Tunis, Tunis El Manar University, 15 Jebel Lakhdhar Street, La Rabta, 1007 Tunis, Tunisia; ^2^National Institute Mongi Ben Hmida of Neurology, Department of Child Neurology, UR12SP24 Abnormal Movements of Neurologic Diseases, Jebel Lakhdhar Street, La Rabta, 1007 Tunis, Tunisia

## Abstract

**Background:**

Epilepsy is one of the most common neurological disorders with about 30% treatment failure rate. An interindividual variations in efficacy of antiepileptic drugs (AEDs) make the treatment of epilepsy challenging, which can be attributed to genetic factors such as ATP-Binding Cassette sub-family B, member1 (*ABCB1*) gene polymorphisms.

**Objective:**

The main objective of the present study is to evaluate the association of *ABCB1* C1236T, G2677T, and C3435T polymorphisms with treatment response among Tunisian epileptic patients.

**Materials and Methods:**

One hundred epileptic patients, originated from north of Tunisia, were recruited and categorized into 50 drug-resistant and 50 drug-responsive patients treated with antiepileptic drugs (AEDs) as per the International League Against Epilepsy. DNA of patients was extracted and *ABCB1* gene polymorphisms studied using polymerase chain reaction-restriction fragment length polymorphism (PCR-RFLP) method.

**Results:**

The C1236T, G2677T, and C3435T polymorphisms were involved into AED resistance. Significant genotypic (C1236T TT (*p* ≤ 0.001); G2677T TT (*p* = 0.001); C3435T TT (*p* ≤ 0.001)) and allelic associations (C1236T T (3.650, *p* ≤ 0.001); G2677TT (1.801, *p* = 0.044); C3435T T (4.730, *p* ≤ 0.001)) with drug resistance epilepsy (DRE) were observed. A significant level of linkage disequilibrium (LD) was also noted between *ABCB1* polymorphisms. Patients with the haplotypes CT and TT (C1236T-G2677T); GT, TC, and TT (G2677T-C3435T); CT and TT (C1236T-C3435T); CTT, TTC, TGT, and TTT (C1236T-G2677T-C3435T) were also significantly associated to AED resistance.

**Conclusions:**

The response to antiepileptics seems to be modulated by TT genotypes, T alleles, and the predicted haplotypes for the tested SNPs in our population. Genetic analysis is a valuable tool for predicting treatment response and thus will contribute to personalized medicine for Tunisian epileptic patients.

## 1. Introduction

Epilepsy is one of the prevalent serious neurological disorders [1] affecting approximately 50 million people worldwide [2].

During the last years, a large variety of antiepileptic drugs (AEDs) with different mechanisms of action were developed, which makes the epilepsy treatment a big challenge [3–5]. In fact, at least one-third of epileptic patients are or become resistant to treatment and experience recurrent seizures [6, 7]. This pharmacoresistance depends on several factors primarily age, epileptic etiology, type, and syndrome, AEDs [8].

Moreover, genetic factors play an important role in the development of refractory epilepsy. Indeed, the prediction of the individual's response to AEDs is very helpful for knowing drug resistance mechanisms which will allow the selection of the appropriate type of drug treatment and early epilepsy surgical evaluation. Several studies focused on identifying the potential genetic markers affecting the pharmacoresistance. They provided several genetic variations affecting pharmacokinetics or pharmacodynamics of AEDs in the treatment of epilepsy. These research works also evaluated the association between variations in drug transporter and their target receptor genes and the occurrence of refractory seizures [9, 10].

One of the best studied drug transporters is the transmembrane P-glycoprotein (P-gp). This ATP-dependent efflux-pump protein ensuring the transport and elimination of diverse AEDs at the blood-brain barrier (BBB) is expressed in the brain (astrocytes, endothelial cells, and neurons) [11–16]. P-gp overexpression reduces the AEDs bioavailability in the epileptic cells, which contributes to refractory epilepsy. Some studies suggested that its altered function could be a result of genetic variants especially SNPs located in the *ABCB1* gene [4, 17–25]. The most studied SNPs in this gene are C1236T (rs1128503) in exon 12, G2677T (rs2032582) in exon 21, and C3435T (rs1045642) in exon 26 [26–32].

The C3435T is commonly considered as a critical SNP in AED resistance [33–36]. Results of assessing the association of *ABCB1* polymorphisms with the resistance to AEDs are discordant. An initial study reported that patients with drug resistance epilepsy (DRE), compared to AED responders, were more likely to have CC genotype (27.5% vs. 15.7%, respectively) than TT genotype (19.5% vs. 29.6%, respectively) [37]. This finding was confirmed in some studies [21, 27, 38, 39], while others showed an opposite result [29, 40–42]. Likewise, other studies [6, 43–47] and meta-analyses revealed no significant association between genetic profile and refractory epilepsy [35, 48–53]. These conflicting findings are essentially due to heterogeneity, phenotyping and genotyping errors, bias, etc.

Despite the fact that there was a significant interest in showing the associations between drug resistance and *ABCB1* 3435 genotypes, the clinical practice of a *ABCB1* SNPs routine testing to predict the patient's response to the therapy has not been yet examined [32, 49, 54]. Therefore, we focus, in this work, on evaluating the relationship between the *ABCB1* C1236T, G2677T, and C3435T polymorphisms and the pharmacoresistant epilepsy in Tunisian patients.

## 2. Materials and Methods

### 2.1. Study Population

The present study includes 100 Tunisian epileptic patients, originated from north of Tunisia (56 males and 44 females) with a mean age of 6.710 ± 4.358. All epilepsy patients were evaluated in the Neuro-pediatric Department at Mongi Ben Hamida National Institute of Neurology. They were diagnosed for epilepsy after a follow-up of one year or more and treated by AED monotherapy or bitherapy.

This study was conducted in accordance with the ethical standards of the declaration of Helsinki [55]. It was approved by a local human research ethics committee (HTHEC-2016-30). An informed consent was signed by all patients and/or their parents.

All subjects were examined by a qualified epilepsy neurologist and had a confirmed diagnosis based on the operational clinical definition of epilepsy [8] and classified according to the guidelines specified in the International League Against Epilepsy (ILAE) [56, 57].

Information on demographic and clinical characteristics were obtained from structured questionnaires and medical records database. The collected information included sex, age, age at seizure onset, family history of epilepsy, types and etiology of seizures, epileptic syndromes, treatment therapy, and the number of prescribed AEDs (Table 1).

#### 2.1.1. Definition of Drug Resistance

According to the definition set by the ILAE, patients were considered as drug-resistant to epilepsy if the adequate trials of two tolerated and appropriately used AED schedules (whether monotherapies or combination) failed to achieve sustained seizure freedom [58].

The nonresponders must not have a lesional pharmacoresistant epilepsy [42, 59, 60].

All associated pathologies that might promote the occurrence of epileptic seizures and may lead to wrong diagnosis of epilepsy were excluded from this study (imaging abnormalities including tumor, progressive or degenerative neurological or systemic disorders, tuberculoma, multiple neurocysticercosis, vascular malformations, and atrophic lesions; hepatic, renal [42, 59, 60], gross neurological deficits (mental retardation, motor/speech), diabetes mellitus [60], hematopoietic [61], cardiac insufficiency [42, 62]; infectious, traumatic, metabolic, and deep psychiatric disorders [62]; cancers [42] or secondary metastases [31]).

On the other hand, any subject who neglected the treatment regimen or presented any exclusion criteria (verified poor compliance by performing blood tests of MAEs [63]; adverse drug reactions of AEDs [61]; alcohol, addiction [59], or drug intake (inducers or inhibitors of enzymes that metabolize MAEs; substrates or inhibitors of P-gp [63]), was also excluded from this work.

#### 2.1.2. Definition of Drug Responsiveness

The patients were considered as drug-responsive if they did not have any type of seizures for, at least, 1 year during AED treatment [58, 64].

In our population, 50 drug-responsive patients constituting “the control group” (males : females = 28 : 22) were matched according to sex, age, and geographic region [42] with 50 drug-resistant patients constituting “the patient group” (males : females = 28 : 22). Refractory and responsive patients were unrelated (Table 1).

### 2.2. DNA Extraction and Genetic Analysis

Blood samples (5-10 ml) were collected in ethylenediamine tetraacetic acid (EDTA) tubes from each patient and control. Genomic DNA was isolated from whole blood samples. For all patients and controls, 3 SNPs of *ABCB1* gene (C1236T, G2677T, and C3435T) were genotyped by polymerase chain reaction (PCR) assay using a SimpliAmp™ (Applied Biosystems-Life Technologies) followed by restriction fragment length polymorphism (RFLP) analysis. The fragments were amplified with 0.4 mM dNTPs, 4 mM MgCl2, nuclease-free water, reaction buffer, 20 *μ*mol/l of primers, and 0.05 U Taq polymerase in a final volume of 50 *μ*l using a PCR Master Mix (Thermo Fisher Scientific) using the following program for the 3 SNPs (rs1128503, rs2032582, rs1045642): 94°C for 2 min, 35 cycles (94°C for 30s, 60°C for 30s, 72°C for 30s), and 72°C for 7 min [65] (Table 2).

After amplification, PCR products were digested using 2 *μ*l specific restriction endonucleases (HaeIII, BanI, and Sau3A1 (Thermo Fisher Scientific)), 9.5 *μ*l nuclease-free water, 4.5 *μ*l restriction enzyme buffer, and 4 *μ*l PCR products in a final volume of 20 *μ*l. The separated fragments were visualized on 3% agarose gel after incubation at 37°C for 16 h. The restriction specific sites and sizes of digested fragments are summarized in Table 3.

### 2.3. Statistical Analysis

The chi-square (*X*^2^) test (2 × 2 contingency tables) was performed to compare allelic and genotypic distribution of *ABCB1* C1236T, G2677T, and C3435T polymorphisms between drug-resistant group (patients group) and drug-responsive group (control group). The association is statistically significant when *p*-value is ≤0.05 [66]. The statistical analysis was conducted with logiciel Epi info™ 7 [67].

Linkage disequilibrium (LD) analysis and haplotype construction were carried out by SHEsis online software [68]. The *r*^2^ (correlation index) and *D* (LD coefficient) were calculated, to test the LD among the 3 loci. If *r*^2^ and ∣*D*′∣ = 1, the alleles are in a complete LD (separated by recombination). If *r*^2^ and ∣*D*′∣ < 1, the LD is disrupted. Associations between allelic, genotype, haplotype, and drug response were estimated by odds ratio (OR) with 95% confidence interval (CI).

## 3. Results

### 3.1. Demographics and Clinic Characteristics

The sex ratio was predominantly male (56 vs. 44%) with a mean age in years of 6.710 ± 4.358 and a mean age at seizure onset of 3.820 ± 3.362.

For patients group, the mean age was 6.220 ± 4.432 vs. 7.200 ± 4.271 for control one.

The mean age at seizure onset for nonresponders is 2.680 ± 2.470 and 4.960 ± 3.752 for responders.

Overall, the most common seizure type was generalized (75%). Focal and focal to bilateral tonic-clonic seizures were minoritary (20% and 5%, respectively).

In the drug-resistant group, 84% patients presented generalized seizures, 6% focal and 10% focal to bilateral tonic-clonic ones, whereas in the drug-responder group, 66% of patients presented generalized and 34% of focal seizures, but no focal to bilateral tonic-clonic seizures were observed.

The syndromes (such as absences, continuous spikes, and waves during sleep (CSWS), Lennox-Gastaut...) constituted only 26% of our epileptic patients: 38% in drug-resistant group vs. 14% in drug-responsive group.

The epileptic etiology for the epileptic patients was mainly unknown (57%). The main factor seems to be genetic (48%) for the drug-resistant patients with known etiology, whereas it seems to be a structural one (20%) for the drug-responsive patients.

The medical history observed is low (16%) in epileptic patients, drug responders, and none.

The patients enrolled in this study received mainly a polytherapy AEDs (42%) as well in drug-resistant patients (84%), while for the drug-responsive patients, it was a monotherapy (68%).

### 3.2. Polymorphisms Analysis and Susceptibility to DRE

#### 3.2.1. Genotypic and Allelic Analysis

**Figure 1:**
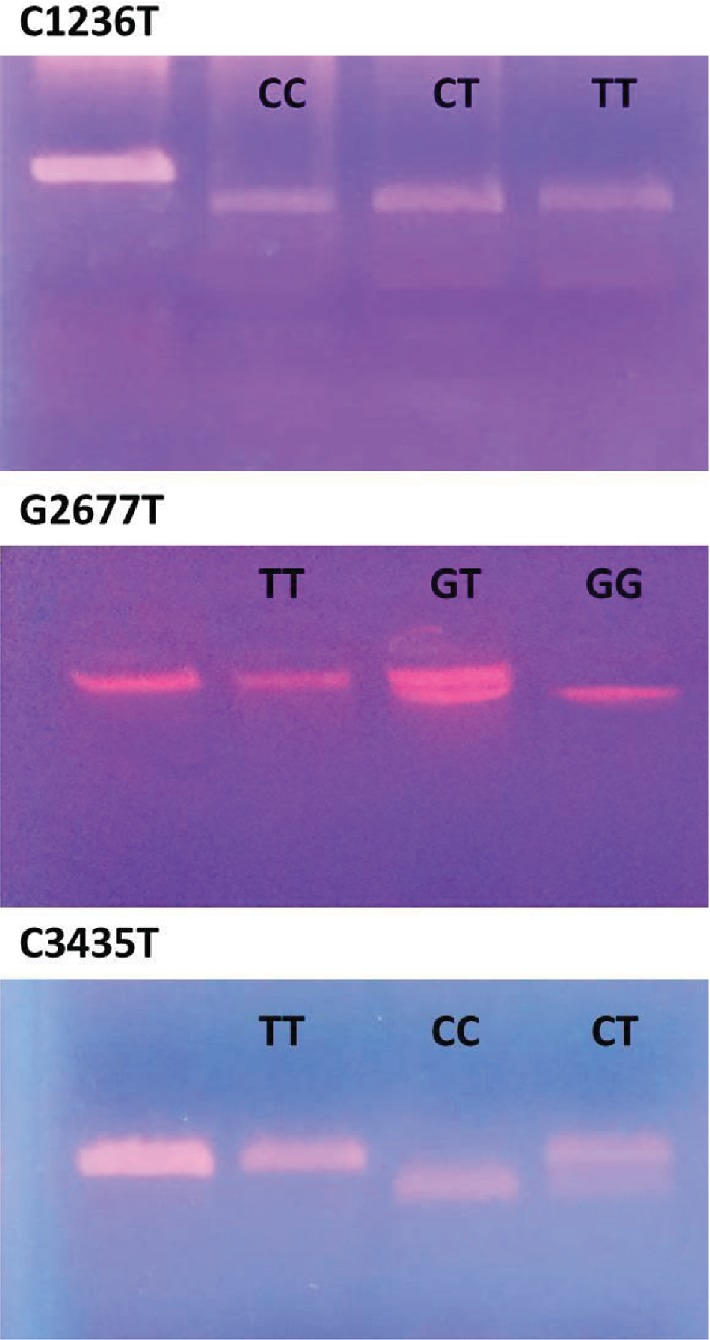
Results of digestion for the three SNPs. For C1236T, well 1: PCR product, well 2: homozygous wild-type CC, well 3: heterozygous CT, and well 4: homozygous mutant type TT. For G2677T, well 1: PCR product, well 2: homozygous mutant-type TT, well 3: heterozygous CT, and well 4: homozygous wild-type GG. For C3435T, well 1: PCR product, well 2: homozygous mutant-type TT, well 3: homozygous wild-type CC, and well 4: heterozygous CT.

We found a significant allelic and genotypic association between C1236T, G2677T, and C3435T polymorphisms and response to AEDs (Figure 1). In fact, we observed that the TT genotypes and (*p* ≤ 0.001 for C1236T, *p* = 0.001 for G2677T, *p* ≤ 0.001 for C3435T) the T allele of the 3 SNPs (T vs. C, OR = 3.650, 2.029-6.563, *p* ≤ 0.001 for C1236T; T vs. C, OR = 1.801, 1.016-3.192, *p* = 0.044 for G2677T; T vs. C, OR = 4.730, 2.604-8.591, *p* ≤ 0.001 for C3435T) were significantly more frequent in drug-resistant patients than in drug-responsive patients. The *ABCB1* genotype and allele frequencies are shown in Tables 4 and 5, respectively.

#### 3.2.2. Haplotypic Analysis

The obtained results showed a significant degree of LD between C1236T and G2677T (∣*D*′∣ = 0.211), G2677T and C3435T (∣*D*′∣ = 0.035), and C1236T and C3435T (∣*D*′∣ = 0.236). In fact, the *r*^2^ coefficient between C1236T and G2677T, G2677T and C3435T, and C1236T and C3435T were 0.033, 0.001, and 0.039, respectively.

The ORs of CT and TT haplotypes (C1236T and G2677T); GT, TC, and TT haplotypes (G2677T and C3435T); and CT and TT haplotypes (C1236T and C3435T) were significantly higher in nonresponder patients than in responder patients: 3.500, 1.152-10.633, *p* = 0.027 and 19.056, 2.395-151.604, *p* = 0.005; 3.778, 1.343-10.628, *p* = 0.012, 2.852, 0.995-8.174, *p* = 0.051, and 36.360, 2.095-631.209, *p* = 0.014; 4.929, 1.503-16.158, *p* = 0.009 and 10.286, 2.209-47.902, *p* = 0.003. The more significant OR was observed in TT haplotype for the 3 combinations of SNPs.

Compared to CGC haplotype, ORs of the association between CTT, TGT, TTC, and TTT haplotypes and drug refractory were 17.414, 0.967-313.749, *p* = 0.053; 5.268, 1.077-25.780, *p* = 0.040; 9.333, 1.121-77.707, *p* = 0.039; 18.910, 1.061-337.144, *p* = 0.046, respectively. The most significant effect was noted in TTT haplotype. The distribution of all *ABCB1* haplotypes is represented in Tables 6–9.

### 3.3. Polymorphisms and Patient Data Correlation

We further correlated separately each collected data factor with each genotype, allele, and haplotypes for the studied SNPs (all significant associations are shown in Tables 10–15).

#### 3.3.1. *ABCB1* Polymorphisms and Gender

After a gender-based stratification, a significant association between male patients and C1236T, G2677T, and C3435T TT genotypes, C1236T and C3435T T alleles, and TT (G2677T-C3435T) haplotype was observed. We also noted an important association between female patients and G2677T and C3435TTT genotypes (Table 10).

#### 3.3.2. *ABCB1* Polymorphisms and Generalized/Focal Epilepsy

The distribution according to the epileptic etiology led to a significant association between generalized seizures and C1236T, G2677T, and C3435T TT genotypes, C1236T and G2677TT alleles, and TG, TT (C1236T-G2677T), GT, TT (G2677T-C3435T), and TC, TT (C1236T-C3435T) and TGT (C1236T-G2677T-C3435T) haplotypes. In contrast, a significant association was obtained between focal seizures and T allele of C3435T and CT (C1236T-C3435T) haplotype (Table 11).

#### 3.3.3. *ABCB1* Polymorphisms and Epileptic Syndromes

The C1236T, G2677T, and C3435T TT genotypes were considerably higher in nonresponders with epileptic syndromes vs. responders (Table 12).

#### 3.3.4. *ABCB1* Polymorphisms and Unknown/Genetic Epileptic Etiology

Significant associations were noticed between genetic etiology and C1236T, G2677T, and C3435T TT genotypes, C1236T T alleles, and TT (C1236T-G2677T), GT, TT (G2677T-C3435T), and CT, TT (C1236T-C3435T) haplotypes. An association between unknown etiology and G2677T GG, TT genotypes and C3435T T alleles was also observed (Table 13).

#### 3.3.5. *ABCB1* Polymorphisms and Medical History

The association analysis of the *ABCB1* gene revealed that C1236T, G2677T, and C3435T TT genotypes, C1236T T alleles, and TT (C1236T-G2677T) haplotypes were significantly associated with medical history (Table 14).

#### 3.3.6. *ABCB1* Polymorphisms and AED Therapy

We evaluated the association of drug responders and nonresponders with *ABCB1* SNPs according to the individual monotherapy, bitherapy, and polytherapy. We only noted a significant association between bitherapy and G2677T TT genotypes and G2677TT alleles (Table 15).

## 4. Discussion

The response to medications varies greatly from one individual to another [69]. The term “drug resistance” is a commonly encountered complication in clinical practice. The concept of DRE existed since the intake of AEDs and the failure of treatment (persistence of seizures). It was observed that patients became resistant to most or to all broad range AEDs with different mechanisms of action [70]. The reported frequency of nonresponders is approximately 30% [4, 10, 71, 72].

According to the obtained results, males were more likely to develop DRE in our study population. This results is in accordance with other reported studies [73–75].

In accordance with epilepsy type, drug-resistant and drug-responsive patients showed a predominance of generalized seizures (84% vs. 66%), compared to focal (6% vs. 34%) and focal to bilateral tonic-clonic seizures (10% vs. 0%). Some studies provided the same results [74, 76, 77], while many others showed the opposite [32, 73, 75, 78–82] due to some changes recently made in the classifications of epilepsies taken into account the type of onset seizures [32, 56]. Epileptic syndromes represented only 26% of our epileptic patients, in fact that most of our patients with isolated seizures have no EEG results or have an EEG with no specific electroclinical syndrome. This result is similar to another study from the center of Tunisia (18.3%) [32]. The etiology remained unknown in 57% of our cases, in drug-resistant patients and drug-responsive ones (52% and 62%). The same result was observed in the study of Banerjee et al. [74].

Medical history was heterogeneous (not only epileptic seizures but other different types of medical histories) and low for epileptic patients and the 2 other groups. It was also observed that the epilepsy drug resistance was frequent in patients treated with more than two AED molecules (84%), while drug-responsive patients responded more to monotherapy treatment (68%). Ajmi et al. [32] have shown the same results as ours except for the result of the polytherapy because they enrolled in their study only patients treated with first-generation AEDs.

As it was previously indicated, the development of DRE depends on several factors [70] such as the genetic differences between individuals. In previous works, the polymorphisms of the *ABCB1* gene, encoding P-gp, were extensively examined in patients with DRE but with conflicting results.

In the present study, we noted a significant association of *ABCB1* C3435T polymorphism with drug resistance in epilepsy. In fact, epileptic seizure recurrence was higher in nonresponders with TT genotype and T allele in comparison with responders.

These findings corroborate those obtained by some studies showing that TT genotype [21, 29, 40, 41] and T allele [71, 72] play an important role in seizure recurrence in drug-resistant patients compared to drug-responsive patients. However, Siddiqui et al. [37] were the first to investigate the association between C3435T polymorphism and refractory epilepsy in 315 Caucasians. They reported that DRE might be genetically determined. In fact, they proved that patients with drug-resistant epilepsy are more likely to have the CC genotype than the TT genotype (OR = 2.66; 95% CI (1.32-5.38), *p* = 0.006). The same results were mentioned in other studies demonstrating a higher percentage of C3435T CC genotype [33, 83] and C allele [84] carriers in nonresponders to AEDs. The study of Ajmi et al. [32] from the center of Tunisia found a significant association between CT, TT genotypes, and DRE. On the other hand, several works and meta-analyses could not establish an association between C3435T polymorphism and resistant epilepsy [30, 31, 40, 45, 49, 71, 85, 86].

The P-gp is mainly found at BBB and in various normal tissues with excretory functions. It plays a central role in the transport of the planar lipophilic agents (AEDs), whose majority are *ABCB1*gene substrates. Considering the presence of some genetic variations in this gene, *ABCB1* overexpression may lead to prevent AEDs from attaining the sites of action and to have the therapeutic effect.

Even if the common genetic variant C3435T is a silent polymorphism that does not alter the amino acid sequence of P-gp, it may influence the transport and the distribution of AEDs, reducing the levels of AEDs in the brain and leading to refractory epilepsy.

It was hypothesized that the CC genotype is associated with overexpression of P-gp near the epileptogenic brain foci [37]. This overexpression together with other efflux transporters in the cerebrovascular endothelium may cause DRE [21].

On the other hand, other studies demonstrated that TT genotype is crucial in P-gp activity influencing the oral bioavailability at the BBB, which based on the assumption that patients bearing this genotype could be more resistant to treatment than those bearing CC genotype [37]. Similar results implied that T allele ensures the overexpression of *ABCB1* in endothelial cells [54, 70, 87].

Thereby, the presence of C3435T SNP may lead to a high P-gp expression in endothelium tissues and in neurons of epileptic patients [18], resulting in pharmacoresistant epilepsy which can be treated by surgery [21, 88]. However, other studies failed to confirm that C3435T SNP is associated to altered P-gp molecular expression and functional activity [32].

The C1236T polymorphism seems likewise to influence the response to AEDs. In fact, we found a significant association between the C1236T TT genotype or T allele and the resistance to AEDs. Contrariwise, the results in the study of Siddiqui et al. [37] showed that the proportion of CC genotype in nonresponders was significantly higher than that in responders (27.5 and 15.7%, respectively), but the proportion of TT genotype was significantly lower in nonresponders than that in responders (19.5 and 29.6%, respectively).

Li et al. [89] failed to find any association in 6324 drug-responsive vs. 6083 drug-resistant patients. The same results were published by Ajmi et al. [32]. As a silent C1236T SNP, no report has until now investigated its possible effect on P-gb activity [32].

A number of studies showed the relation between the variation of the ABCB1 gene expression and/or the P-gp activities and *ABCB1* G2677T (Ala893Thr) SNP [18, 21, 27]. Our results demonstrated that the drug-resistant patients are more likely to have the TT genotype than the GG genotype. These findings confirm those presented in the study of Seo et al. [29] showing that the risk of drug resistance was more significant in Japanese patients with the TT genotype than those with the GG genotype. In the study of Ajmi et al. [32], the GT and TT genotypes were present in patients with DRE. However, these results contrast with those obtained in other works. For instance, Sánchez et al. [90] found that the distribution of the GG genotype was more higher in the Caucasian adults drug-resistant than drug-responsive.

In the meta-analysis of Li et al. [89], no association was observed in Asians and Caucasians. Overall, serine/alanine amino acid of the coding polymorphism increases P-gp activity, even so other studies failed to demonstrate this effect on P-gp intracellular location, expression level, and function [32].

The discordant results of all these studies could be explained by [91, 92]:

- The small sizes of the studied populations [63]
- The heterogeneity in selection criteria for study populations (different used study designs and subject definitions, recurrent epileptic seizures, variation in duration which precedes the identification of drug resistance...) [63]
- The variable methodologies applied for phenotyping and genotyping [63]
- The association between *ABCB1* C3435T polymorphism and AEDs might be not real [93, 94]
- The different ethnicities of patients may be correlated to the modification the P-gp expression. Ajmi et al. [32] reported that the level of expression of the T allele of the most studied SNP *ABCB1* C3435T in DRE varied from one ethnic group to another. Moreover, its lowest frequencies were found in Tunisian (0.2) and Egyptian (0.3) [95] populations compared to the other ethnicities (Chinese and Iranian)
- The heterogeneity of epilepsy including multiple syndromes with various etiologies [40, 95, 96]
- The different other actors, such as age, various epileptic etiologies, and variability in drug response to a large AEDs number, should be taken into account

Indeed, Sánchez et al. [90] found a lower risk associated with *ABCB1* 3435TT or 2677TT genotypes in the subgroup of patients (>12 years). Nevertheless, drug-resistant and drug-responsive groups had different origins of epilepsy and treatments [93, 94]. The most used AEDs were P-gp substrates (phenytoin and phenobarbital) in adults with symptomatic epilepsy (Engel classification). On the other hand, the most employed AED in the patients (<12 years) with idiopathic epilepsy was a nonsubstrate P-gp (VPA) due to the fact that CBZ and VPA have not been yet reported to be a drug substrates of P-gp [97].

Thus, it becomes necessary to confirm the association between *ABCB1* polymorphisms and the levels of P-gp expression and activity in brain tissue in patients with refractory epilepsy before admitting the role of SNPs in resistance to MAEs [98]. So far, no conclusive evidence of C3435T-dependent P-gp expression at the BBB level has substantiated the relationship between *ABCB1* polymorphisms and the expression levels of *ABCB1* brain mRNA or P-gp proteins in refractory epilepsy [6, 98–100].

Haplotypic analysis indicated that CT and TT haplotypes (C1236T and G2677T) were significantly higher in patients with DRE. The GT, TC, and TT haplotypes (G2677T and C3435T) increased considerably the risk of drug-resistant epilepsy. Only the TT haplotype was shown in 3 other studies [30, 32, 39]. Nevertheless, no association was observed in other population [101]. The patients with DRE were more likely to have CT and TT haplotypes (C1236T and C3435T), compared to drug-responsive patients.

A significant level LD was observed between the C1236T, G2677T, and C3435T SNPs, indicating that these loci reacted as a complex haplotypic system. The haplotype combination CTT, TGT, TTC, and TTT was significantly associated with poor response, while the haplotype combination of CGC was related to good drug response. However, Siddiqui et al. [37] as well as Zimprich et al. [27] showed that the resistance to AEDs therapy was significantly influenced by the presence of the CGC haplotype in Caucasians.

The haplotypic analysis, in another study, [28] demonstrated that Asians with the CGC, TGC, and TTT haplotypes were more likely to be drug resistant. Other studies failed to report any significant association between haplotypes and DRE [29, 30, 102]. As an example, a meta-analysis for haplotype that included a total of 26 publications (*n* = 7,831 patients) did not reveal any significant associations between polymorphisms and their haplotypes and the response to AEDs whether in the general population or in ethnic subgroups. In addition, the data available in this meta-analysis did not allow carrying out subgroup analyses by the used types of AEDs or epilepsy [35].

Some major reported factors, such as the complex haplotype system, the low sample size, the clinical and genetic heterogeneity in epilepsy, and the environment [29, 35], could explain these contradictory results.

Based on our findings, the importance of *ABCB1* haplotype system was highlighted. Screening for these SNPs, which are in high LD, could be related to a significant decrease in intracellular substrate concentration leading to the P-gp overactivity in the BBB [89, 103–107] and could be a stronger marker. Therefore, the combinations of bi- and triallelic haplotypes should be studied.

As in the most incidence and prevalence studies of epilepsy, the disease is more frequent in male than in female patients [108–110] due to the fact that the majority of men refuse to marry women with epilepsy, which leads to a strong concealment of the pathology.

Nevertheless, we found significant results in female patients with DRE. Indeed, Sidenvall et al. [111] reported that the incidence of infantile epilepsy was rather raised in girls than in boys.

Another study reported that there is no difference between the two sexes as far as DRE is concerned [112].

We also found an increased frequency of focal epilepsies. The same results were obtained in many other works [32, 78, 79, 81, 82]. We observed a more significant predominance of generalized seizures compared to focal seizures, in our study. Opposite findings were found in others which classified patients based on of the previous classification [74, 76, 77]. It is important to notice that recently some changes were made on the classification of epilepsies which include the beginning of seizures. This update was taken into account for our study.

Epileptic syndromes are determined by clinical context, epileptic symptoms, and EEG abnormalities. We noted a limited frequency of resistant patients with epileptic syndromes (38%) due to the lack of access to the EEG. On the other hand, significant associations were found in genotypes.

Obviously, we found that the unknown etiology was also important, which concords to the study of Banerjee et al. [74], reviewing many studies reporting a preponderance of seizures of unknown cause. In addition, the genetic research has evolved to identify multiple genes and genetic variations in epileptic patients, which in turn has led to very significant results concerning genetic etiology (48%) in our drug-resistant patients.

We also noted a significant association between the DRE and the medical history. Indeed, different medical histories may occur in epileptic patients, which does not necessarily explain the resistance to AEDs. According to Hitiris et al. [113], the risk of developing DRE is more important with stroke history or severe trauma. However, in our study, resistant patients did not have a lesional DRE contrarily to the study of Ajmi et al. [32] where the frequency of structural etiology was more common.

Finally, patients enrolled in our study were resistant to all the different administered AEDs. The stratification by each type of AEDs was useless because it can distort results. Our results showed that only bitherapy seems to be concerned by the association of resistance with genetic biomarkers. The same result was showed in the studies of Ajmi et al. [32] and Kwan and Brodie [114]. They noted that the patients who could be predisposed to refractory epilepsy justify the need of a bitherapy.

## 5. Conclusion

Epilepsy was intensively studied in a large number of research works that used pharmacogenetics in an attempt to improve the therapeutic response relying on a personalized therapy. We found that *ABCB1* polymorphism increases the risk of developing AED resistance. The obtained findings support in part those provided by previous Tunisian study. The screening for the detection of these polymorphisms may be an effective method to a better therapeutic management of the epilepsy by choosing the best treatment option for each patient and predicting the treatment outcome of the newly diagnosed Tunisian patients before the administration of medication. This will, in the long term, reduce the morbidity among them.

## Figures and Tables

**Table 1:** General characteristics of the study population.

Variables		Epileptic patients (*N* = 100)		Patient group (*N* = 50)		Control group (*N* = 50)	
		*n*	%	*n*	%	*n*	%
Sex ratio	Male	56	56	28	56	28	56
	Female	44	44	22	44	22	44

Age (years)		6.710 ± 4.358		6.220 ± 4.432		7.200 ± 4.271	

Age at seizure onset (years)		3.820 ± 3.362		2.680 ± 2.470		4.960 ± 3.752	

Type of seizure	Generalized	75	75	42	84	33	66
	Focal	20	20	3	6	17	34
	Focal to bilateral tonic-clonic	5	5	5	10	0	0

Epileptic syndrome	Yes	26	26	19^a^	38	7^b^	14
	No	74	74	31	62	43	86

Etiology of epilepsy	Autoimmune	0	0	0	0	0	0
	Genetic	31	31	24	48	7	14
	Infectious	1	1	0	0	1	2
	Metabolic	1	1	0	0	1	2
	Structural	10	10	0	0	10	20
	Unknown	57	57	26	52	31	62

Medical history	Yes	16	16	8^c^	16	8^d^	16
	No	84	84	42	84	42	84

Antiepileptic treatment	Monotherapy	34	34	0	0	34^e^	68
	Bitherapy	24	24	8^f^	16	16^g^	32
	Polytherapy	42	42	42^h^	84	0	0

Values (*n* and %). ^a^Absences, Angelman, continuous spikes and waves during sleep (CSWS), Dalla Benardina, Doose, Dravet, Ohtahara, early myoclonic encephalopathy (EME), generalized epilepsy with febrile seizures plus (GEFS+), juvenile myoclonic epilepsy (JME), Lennox-Gastaut, WEST. ^b^Absences, benign epilepsy with centro-temporal spikes (BECTS), early myoclonic encephalopathy (EME), idiopathic generalized epilepsies (IGE), tuberous sclerosis of Bourneville (STB). ^c^Appendectomy, bronchopneumopathy, dehydration and gastroenteritis, febrile seizures, mental retardation with behavioral disorder, neonatal cyanosis, *recurrent bilateral otitis media*, recurrent urinary tract infections, varicella ^d^Appendectomy, bilateral hernia, bilateral testicular ectopia, enuresis, febrile seizures, gastroesophageal reflux, trauma, maternal-feotal infection, mumps, neonatal hypoglycemia, strabismus, stunting delay. ^e^Controls were prescribed single AED (carbamazepine or valproic acid). ^f^Patients were prescribed a combination of 2 AEDs (carbamazepine, clonazepam, lamotrigine, phenobarbital, valproic acid, vigabatrin). ^g^Controls were prescribed a combination of 2 AEDs (carbamazepine, clonazepam, phenobarbital, valproic acid). ^h^Patients were prescribed a combination of diverse AEDs (carbamazepine, clobazam, clonazepam, diazepam, ethosuximide, lamotrigine, levetiracetam, phenobarbital, phenytoin, topiramate, valproic acid, vigabatrin).

**Table 2:** Sequences of F and R primers used in the study, *T*_m_, and size of the amplicons for each SNP [65].

SNP	Exon	Primer F	Primer R	Tm (°C)	Amplicon size (bp)
*ABCB1* C1236T	12	TATCCTGTGTCTGTGAATTGCC	CCTGACTCACCACACCAATG	60	366
*ABCB1* G2677T	21	TGCAGGCTATAGGTTCCAGG	TTTAGTTTGACTCACCTTCCCG	60	224
*ABCB1* C3435T	26	TGTTTTCAGCTGCTTGATGG	AAGGCATGTATGTTGGCCTC	60	197

F: forward; R: reverse; *T*_*m*_: melting temperature; bp: base pair.

**Table 3:** Size and restriction recognition sites of digested fragments for each SNP [65].

SNP	Enzyme^∗^	Unit size^∗^(U)	Restriction site^∗^	Size of digested fragment (bp)
*ABCB1* C1236T	HaeIII (BsuRI)	3000	5′…GG⬇CC…3′ 3′…CC⬆GG…5′	Wild type: 269 + 62 + 35
				Mutated type: 269 + 97

*ABCB1* G2677T	BanI (BshNI)	2000	5′…G⬇GYRCC…3′ 3′…CCRYG⬆G…5′	Wild type: 198 + 26
				Mutated type: 224

*ABCB1* C3435T	Sau3A1 (Bsp143I)	1500	5′…⬇GATC…3′ 3′…CTAG⬆…5′	Wild type: 158 + 39
				Mutated type: 197

^∗^ https://www.thermofisher.com/tn/en/home.html; U: units; bp: base pair.

**Table 4:** Distribution of *ABCB1* genotypes frequencies in drug-responsive and drug-resistant patients.

SNP	*ABCB1* genotype	Drug-resistant patients (*N* = 50)		Drug-responsive patients (*N* = 50)		ORs	95% CI	*p* value	*X* ^2^
		*n*	%	*n*	%				
*ABCB1* C1236T	CC vs. CT+TT	6	12	17	34	0.265	0.094-0.745	0 .012	6.830
	CT vs. CC+TT	18	56	27	54	0.479	0.215-1.068	0.072	3.270
	**TT vs. CC+CT**	26	52	6	12	**7.944**	**2.872-21.978**	**≤0.001**	**18.380**
	**TT vs. CC**	26	52	6	12	**12.278**	**3.393-44.433**	**≤0.001**	**16.740**
	CT vs. CC	18	56	27	54	1.889	0.625-5.705	0.260	2.890
	**TT vs. CT**	26	52	6	12	**6.500**	**2.231-18.940**	**≤0.001**	**12.990**

*ABCB1* G2677T	GG vs. GT+TT	10	20	3	6	3.917	1.008-15.220	0.049	4.330
	GT vs. GG+TT	13	26	41	82	0.077	0.030-0.201	≤0.001	31.560
	**TT vs. GG+GT**	27	54	6	12	**8.609**	**3.110-23.832**	**≤0.001**	**19.950**
	TT vs. GG	27	54	6	12	1.350	0.282-6.453	0.707	0.140
	GT vs. GG	13	26	41	82	0.095	0.023-0.399	0.001	12.980
	**TT vs. GT**	27	54	6	12	**14.192**	**4.808-41.895**	**0.001**	**27.500**

*ABCB1* C3435T	CC vs. CT+TT	11	22	24	48	0.306	0.128-0.729	0.008	7.430
	CT vs. CC+TT	9	18	20	40	0.329	0.132-0.824	0.018	5.880
	**TT vs. CC+CT**	30	60	6	12	**11**	**3.952-30.614**	**≤0.001**	**25**
	**TT vs. CC**	30	60	6	12	**10.909**	**3.523-33.782**	**≤0.001**	**19.590**
	CT vs. CC	9	18	20	40	0.982	0.339-2.840	0.973	0
	**TT vs. CT**	30	60	6	12	**11.111**	**3.422-36.081**	**≤0.001**	**18.300**

Values (*n* and %). ORs: odds ratio; 95% CI: confidence interval; significant *p* value ≤ 0.05; *X*^2^: chi-square.

**Table 5:** Distribution of *ABCB1* allele frequencies in drug-responsive and drug-resistant patients.

SNP	*ABCB1* allele	Drug-resistant patients (*N* = 50) (%)	Drug-responsive patients (*N* = 50) (%)	ORs	95% CI	*p* value	*X* ^2^
*ABCB1* C1236T	C	30	61	0.274	0.152-0.493	≤0.001	19.380
	**T**	70	39	**3.650**	**2.029-6.563**	**≤0.001**	

*ABCB1* G2677T	G	33	47	0.555	0.313-0.985	0.044	4.080
	**T**	67	53	**1.801**	**1.016-3.192**	**0.044**	

*ABCB1* C3435T	C	31	68	0.211	0.116-0.384	≤0.001	27.380
	**T**	69	32	**4.730**	**2.604-8.591**	**≤0.001**	

Values (*n* and %). ORs: odds ratio; 95% CI: confidence interval; significant *p* value ≤ 0.05; *X*^2^: chi-square.

**Table 6:** Haplotype frequencies of the *ABCB1* C1236T and G2677T polymorphisms in drug-responsive and drug-resistant patients.

*ABCB1* C1236T-G2677T haplotype	Drug-resistant patients (*N* = 50)		Drug-responsive patients (*N* = 50)		ORs	95% CI	*p* value	*X* ^2^
	*n*	%	*n*	%				
CG	10	20	39	78	0.071	0.027-0.185	≤0.001	33.650
CT	14	28	5	10	**3.500**	**1.152-10.633**	**0.027**	**5.260**
TG	12	24	5	10	2.842	0.919-8.791	0.070	3.470
TT	14	28	1	2	**19.056**	**2.395-151.604**	**0.005**	**10.700**

Values (*n* and %). ORs: odds ratio; 95% CI: confidence interval; significant *p* value ≤ 0.05; *X*^2^: chi-square.

**Table 7:** Haplotype frequencies of the *ABCB1* G2677T and C3435T polymorphisms in drug-responsive and drug-resistant patients.

*ABCB1* G2677T-C3435T haplotype	Drug-resistant patients (*N* = 50)		Drug-responsive patients (*N* = 50)		ORs	95% CI	*p* value	*X* ^2^
	*n*	%	*n*	%				
GC	6	12	38	76	0.043	0.015-0.126	≤0.001	41.560
GT	17	34	6	12	**3.779**	**1.343-10.628**	**0.019**	**6.830**
TC	14	28	6	12	**2.852**	**0.995-8.174**	**0.051**	**4**
TT	13	26	0	0	**36.360**	**2.095-631.209**	**0.014**	**14.940**

Values (*n* and %). ORs: odds ratio; 95% CI: confidence interval; significant *p* value ≤ 0.05; *X*^2^: chi-square.

**Table 8:** Haplotype frequencies of the *ABCB1* C1236T and C3435T polymorphisms in drug-responsive and drug-resistant patients.

*ABCB1* C1236T-C3435T haplotype	Drug-resistant patients (*N* = 50)		Drug-responsive patients (*N* = 50)		ORs	95% CI	*p* value	*X* ^2^
	*n*	%	*n*	%				
CC	9	18	40	80	0.055	0.020-0.149	≤0.001	38.460
CT	15	30	4	8	**4.929**	**1.503-16.158**	**0.009**	**7.860**
TC	11	22	4	8	3.244	0.956-11.001	0.059	3.840
TT	15	30	2	4	**10.286**	**2.209-47.902**	**0.003**	**11.980**

Values (*n* and %). ORs: odds ratio; 95% CI: confidence interval; significant *p* value ≤ 0.05; *X*^2^: chi-square.

**Table 9:** Haplotype frequencies of the *ABCB1* C1236T, G2677T, and C3435T polymorphisms in drug-responsive and drug-resistant patients.

*ABCB1* C1236T-G2677T-C3435T haplotype	Drug-resistant patients (*N* = 50)		Drug-responsive patients (*N* = 50)		ORs	95% CI	*p* value	*X* ^2^
	*n*	%	*n*	%				
CGC	3	6	35	70	0.027	0.0073-0.1019	≤0.001	43.460
TTC	8	16	1	2	**9.333**	**1.1210-77.7072**	**0.039**	**5.980**
TGC	1	2	3	6	0.320	0.0321-3.1837	0.331	1.040
CTT	7	14	0	0	**17.414**	**0.9665-313.7492**	**0.053**	**7.530**
CGT	8	16	4	8	2.191	0.6145-7.8082	0.227	1.520
TTT	8	16	0	0	**18.910**	**1.0607-337.1442**	**0.046**	**8.700**
CTC	6	12	5	10	1.227	0.3490-4.3158	0.750	0.100
TGT	9	18	2	4	**5.268**	**1.0766-25.7798**	**0.040**	**5.010**

Values (*n* and %). ORs: odds ratio; 95% CI: confidence interval; significant *p* value ≤ 0.05; *X*^2^: chi-square.

**Table 10:** Association of *ABCB1* polymorphisms and drug resistance in male and female epileptic subgroups.

Male		Drug-resistant patients (*N* = 28)		Drug-responsive patients (*N* = 28)		ORs	95% CI	*p* value	*X* ^2^
		*n*	%	*n*	%				
*ABCB1* C1236T genotypes	TT vs. CC+CT	16	57.143	3	10.714	11.111	2.707-45.613	0.001	13.460
	TT vs. CC	16	84.211	3	25	16.000	2.654-96.472	0.003	10.870
	TT vs. CT	16	64	3	15.789	9.482	2.160-41.612	0.003	10.230
*ABCB1* G2677T genotypes	TT vs. GG+GT	15	53.571	4	14.286	6.923	1.900-25.228	0.003	9.640
*ABCB1* C3435T genotypes	TT vs. CC+CT	18	64.286	4	14.286	10.800	2.912-40.057	≤0.001	14.670
	TT vs. CC	18	78.261	4	22.222	12.600	2.843-55.841	0.001	12.750
*ABCB1* C1236T alleles	T vs. C	41	73.214	22	39.286	4.224	1.901-9.386	≤0.001	13.100
*ABCB1* C3435T alleles	T vs. C	41	73.214	18	32.143	5.770	2.554-13.037	≤0.001	18.950
*ABCB1* G2677T-C3435T	TT	7	25	0	0	19.884	1.076-367.565	0.045	8

Female		Drug-resistant patients (*N* = 22)		Drug-responsive patients (*N* = 22)		ORs	95% CI	*p* value	*X* ^2^
		*n*	%	*n*	%				
*ABCB1* G2677T genotypes	TT vs. GT	12	75	2	9.524	28.500	4.504-180.326	≤0.001	16.550
*ABCB1* C3435T genotypes	TT vs. CT	12	75	2	16.667	15.000	2.258-99.643	0.005	9.330

Values (*n* and %). ORs: odds ratio; 95% CI: confidence interval; significant *p* value ≤ 0.05; *X*^2^: chi-square.

**Table 11:** Association of *ABCB1* polymorphisms and drug resistance in generalized and focal epileptic subgroups.

Generalized		Drug-resistant patients (*N* = 42)		Drug-responsive patients (*N* = 33)		ORs	95% CI	*p* value	*X* ^2^
		*n*	%	*n*	%				
*ABCB1* C1236T genotypes	TT vs. CC+CT	23	54.762	2	6.061	18.763	3.968-88.729	≤0.001	19.720
	TT vs. CC	23	82.143	2	14.286	27.600	4.644-164.027	≤0.001	17.840
*ABCB1* G2677T genotypes	TT vs. GG+GT	24	57.143	4	12.121	7.250	2.209-23.800	0.001	12.410
	TT vs. GT	24	68.571	4	12.903	14.727	4.137-52.423	≤0.001	20.860
*ABCB1* C3435T genotypes	TT vs. CC+CT	26	61.905	3	9.091	16.250	4.254-62.079	≤0.001	21.740
	TT vs. CC	26	72.222	3	16.667	13.000	3.086-54.773	0.001	14.900
	TT vs. CT	26	81.250	3	16.667	21.667	4.717-99.530	≤0.001	19.720
*ABCB1* C1236T alleles	TT vs. CT	23	61.162	2	9.524	15.607	3.147-77.409	0.001	15.140
	T vs. C	60	71.429	23	34.848	4.674	2.337-9.348	≤0.001	20.010
*ABCB1* G2677T alleles	T vs. G	59	70.238	35	53.030	2.090	1.067-4.096	0.032	4.680
*ABCB1* C1236T-G2677T	TG	9	21.429	1	3.030	8.727	1.045-72.888	0.045	5.410
	TT	14	33.333	1	3.030	16.000	1.977-129.518	0.009	10.610
*ABCB1* G2677T-C3435T	GT	13	30.952	3	9.091	4.483	1.156-17.382	0.030	5.260
	TT	12	28.571	0	0	27.459	1.559-483.811	0.024	11.220
*ABCB1* C1236T-C3435T	TC	10	23.810	2	6.061	4.844	0.981-23.908	0.053	4.330
	TT	13	30.952	0	0	30.661	1.746-538.489	0.019	12.360

Focal		Drug-resistant patients (*N* = 3)		Drug-responsive patients (*N* = 17)		ORs	95% CI	*p* value	*X* ^2^
		*n*	%	*n*	%				
*ABCB1* C3435T alleles	T vs. C	5	83.333	11	32.353	10.455	1.087-100.599	0.042	5.520
*ABCB1* C1236T-C3435T	CT	2	66.667	1	5.882	32.000	1.389-737.501	0.030	7.390

Values (*n* and %). ORs: odds ratio; 95% CI: confidence interval; significant *p* value ≤ 0.05; *X*^2^: chi-square.

**Table 12:** Association of *ABCB1* polymorphisms and drug resistance in epileptic syndrome subgroups.

Presence of syndrome		Drug-resistant patients (*N* = 19)		Drug-responsive patients (*N* = 7)		ORs	95% CI	*p* value	*X* ^2^
		*n*	%	*n*	%				
*ABCB1* C1236T genotypes	TT vs. CC+CT	11	57.895	0	0	20.294	1.014-406.357	0.049	7.020
	TT vs. CT	11	69.750	0	0	27.182	1.286-574.353	0.034	8.250
*ABCB1* G2677T genotypes	TT vs. GT	9	64.286	0	0	22.455	1.051-479.955	0.046	7.010
*ABCB1* C3435T genotypes	TT vs. CT	11	91.667	1	20	44.000	2.193-882.709	0.013	8.730

Values (*n* and %). ORs: odds ratio; 95% CI: confidence interval; significant *p* value ≤ 0.05; *X*^2^: chi-square.

**Table 13:** Association of *ABCB1* polymorphisms and drug resistance in unknown and genetic epileptic subgroups.

Genetic		Drug-resistant patients (*N* = 24)		Drug-responsive patients (*N* = 7)		ORs	95% CI	*p* value	*X* ^2^
		*n*	%	*n*	%				
*ABCB1* C1236T genotypes	TT vs. CC+CT	14	58.333	0	0	20.714	1.062-404.123	0.046	7.450
	TT vs. CT	14	70	0	0	24.539	1.175-512.647	0.039	7.950
*ABCB1* G2677T genotypes	TT vs. GT	12	60	0	0	19.118	0.947-386.136	0.054	6.690
*ABCB1* C3435T genotypes	TT vs. CC+CT	16	66.667	1	14.286	12.000	1.226-117.417	0.033	6
	TT vs. CT	16	100	1	20	99.000	3.418-2867.633	0.008	15.810
*ABCB1* C1236T alleles	T vs. C	33	68.750	5	35.714	3.960	1.132-13.850	0.031	4.990

Unknown		Drug-resistant patients (*N* = 26)		Drug-responsive patients (*N* = 31)		ORs	95% CI	*p* value	*X* ^2^
		*n*	%	*n*	%				
*ABCB1* G2677T genotypes	GG vs. GT+TT	6	23.077	1	3.226	9.000	1.006-80.525	0.049	5.170
	TT vs. GG+GT	15	57.692	5	16.129	7.091	2.066-24.344	0.002	10.730
*ABCB1* C3435T genotypes	TT vs. CC	14	82.353	3	16.667	23.333	4.021-135.391	≤0.001	15.100
*ABCB1* C3435T alleles	T vs. C	37	71.154	19	30.645	5.583	2.491-12.513	≤0.001	18.570
*ABCB1* C1236T-G2677T	TT	6	23.077	1	3.226	9.000	1.006-80.525	0.049	5.170
*ABCB1* G2677T-C3435T	TT	6	23.077	0	0	19.976	1.067-374.011	0.045	8
*ABCB1* C1236T-C3435T	CT	8	30.769	2	6.452	6.444	1.229-33.805	0.028	5.780
	TT	6	23.077	1	3.226	9.000	1.006-80.525	0.049	5.170

Values (*n* and %). ORs: odds ratio; 95% CI: confidence interval; significant *p* value ≤ 0.05; *X*^2^: chi-square.

**Table 14:** Association of *ABCB1* polymorphisms and drug resistance in medical history subgroups.

Presence of medical history		Drug-resistant patients (*N* = 10)		Drug-responsive patients (*N* = 11)		ORs	95% CI	*p* value	*X* ^2^
		*n*	%	*n*	%				
*ABCB1* C1236T genotypes	TT vs. CC+CT	6	60	1	9.090	15.000	1.342-167.645	0.028	6.110
	TT vs. CC	6	100	1	20	39.000	1.277-1190.913	0.036	7.540
*ABCB1* G2677T genotypes	TT vs. GG+GT	6	60	1	9.090	15.000	1.342-167.645	0.028	6.110
	TT vs. GT	6	66.667	1	10	18.000	1.496-216.630	0.023	6.540
*ABCB1* C3435T genotypes	TT vs. CC+CT	6	60	1	9.090	15.000	1.342-167.645	0.028	6.110
	TT vs. CT	6	85.714	1	12.5	42.000	2.136-825.760	0.014	8.040
*ABCB1* C1236T alleles	T vs. C	16	80	8	36.364	7.000	1.729-28.337	0.006	8.150
*ABCB1* C1236T-G2677T	TT	5	50	0	0	23.000	1.070-494.601	0.045	7.220

Values (*n* and %). ORs: odds ratio; 95% CI: confidence interval; significant *p* value ≤ 0.05; *X*^2^: chi-square.

**Table 15:** Association of *ABCB1* polymorphisms and drug resistance in epileptic patients with AEDs bitherapy subgroups.

AEDs bitherapy		Drug-resistant patients (*N* = 8)		Drug-responsive patients (*N* = 16)		ORs	95% CI	*p* value	*X* ^2^
		*n*	%	*n*	%				
*ABCB1* G2677T genotypes	TT vs. GG+GT	6	75	2	12.500	21.000	2.372-185.937	0.006	9.380
	TT vs. GT	6	75	2	12.500	21.000	2.372-185.937	0.006	9.380
*ABCB1* G2677T alleles	T vs. G	14	87.500	18	56.250	5.444	1.058-28.011	0.043	4.690

Values (*n* and %). ORs: odds ratio; 95% CI: confidence interval; significant *p* value ≤ 0.05; *X*^2^: chi-square.

## Data Availability

The data underlying the findings of the current study are available from the corresponding author on reasonable request.
